# Genetic Variability in Leishmaniasis-Causing *Leishmania infantum* in Humans and Dogs from North-East Spain

**DOI:** 10.3390/ani14121796

**Published:** 2024-06-15

**Authors:** Xavier Roca-Geronès, Clara Sala, Diana Marteles, Sergio Villanueva-Saz, Cristina Riera, Mª Magdalena Alcover, Roser Fisa

**Affiliations:** 1 Departament de Biologia, Sanitat i Medi Ambient, Secció de Parasitologia, Facultat de Farmàcia i Ciències de l’Alimentació, Universitat de Barcelona, 08028 Barcelona, Spain; 2 Animal Pathology Department, Veterinary Faculty, University of Zaragoza, Miguel Servet 177, 50013 Zaragoza, Spainsvs@unizar.es (S.V.-S.); 3 Clinical Immunology Laboratory, Veterinary Faculty, University of Zaragoza, Miguel Servet 177, 50013 Zaragoza, Spain; 4 Instituto Agroalimentario de Aragón-IA2 (Universidad de Zaragoza), 50013 Zaragoza, Spain

**Keywords:** *Leishmania infantum*, kDNA, intraspecific, genotype, SNP, RFLP

## Abstract

**Simple Summary:**

The aim of this study was to gain new insights into the genetic diversity of the parasite *Leishmania infantum*, which causes leishmaniasis disease in the Mediterranean basin. Twenty-six DNA samples of *L. infantum* obtained from ten hospital patients in Barcelona and five dogs from Aragon in north-east Spain were analyzed to learn more about how the parasite behaves and spreads. The use of two techniques revealed several genetic variations, some of them previously unreported. Single-nucleotide polymorphism analysis identified genotype G13 as the most common, whereas genotype B was the most frequent according to restriction fragment length polymorphism analysis. Both methods indicated that several genotypes were present in both human and dog samples. By highlighting the genetic diversity of this parasite, these results may help to improve tracking and management of the disease. Increasing knowledge of the parasite will allow scientists to develop better strategies to control its spread and protect both humans and animals from infection.

**Abstract:**

*Leishmania infantum* is the primary cause of visceral and cutaneous leishmaniasis in the European Mediterranean region. Subspecies-level characterization of *L. infantum* aids epidemiological studies by offering insights into the evolution and geographical distribution of the parasite and reservoir identity. In this study, conducted in north-east Spain, 26 DNA samples of *L. infantum* were analyzed, comprising 21 from 10 humans and 5 from 5 dogs. Minicircle kinetoplast DNA (kDNA) polymerase chain reaction assays using primers MC1 and MC2, followed by sequencing, were employed to assess intraspecific genetic variability. Single-nucleotide polymorphism (SNP) analysis detected seven genotypes (G1, G2, G12*–G15*, and G17*), with five being reported for the first time (*). The most prevalent was the newly described G13 (54%), while the other currently identified genotypes were predominantly found in single samples. The in silico restriction fragment length polymorphism (RFLP) method revealed five genotypes (B, F, N, P, and W), one of them previously unreported (W). Genotype B was the most prevalent (85%), comprising three SNP genotypes (G1, G2, and G13), whereas the other RFLP genotypes were associated with single SNP genotypes. These kDNA genotyping methods revealed significant intraspecific genetic diversity in *L. infantum*, demonstrating their suitability for fingerprinting and strain monitoring.

## 1. Introduction

Leishmaniasis is a disease often associated with malnutrition and a fragile immune system [1], its clinical manifestations depending on the causative *Leishmania* species and the immune status of the host. The most common form of the disease is cutaneous leishmaniasis (CL), followed by mucocutaneous leishmaniasis and the potentially life-threatening visceral leishmaniasis (VL), also known as kala-azar [1].

In the European Mediterranean basin, *Leishmania infantum* is known to cause human and canine leishmaniasis (HL and CanL) [2], although its incidence is underestimated, especially in CL cases [3,4]. In patients with human immunodeficiency virus (HIV), *L. infantum* is a serious opportunistic parasite, causing VL with high rates of relapse and mortality [1,5]. Cases of VL–HIV coinfection began to emerge in the Mediterranean region in the 1990s, but have now drastically decreased [6].

In Spain, the transmission cycle of *L. infantum* involves the female of two sandfly vectors (*Phlebotomus perniciosus* and *P. ariasi*), with dogs acting as the main reservoir. Humans are considered accidental hosts, and other reservoirs have also been reported [7,8]. A recent study of *L. infantum* infection in apparently healthy dogs in Spain found a seroprevalence rate of 5% [9]. However, variable seroprevalence levels have been detected between regions of Spain, ranging from 13.9% in the Community of Valencia to 6.7% in Aragón and 3.0% in Catalonia [9]. The global seroprevalence in Spain when including both symptomatic and asymptomatic dogs is reported to range between 8.9% and 13.9% [10,11,12,13]. Regarding other domestic animals, epidemiological studies in Spain report highly variable *L. infantum* seroprevalence in cats, ranging from 1.29% to 60% according to the region [7].

With respect to HL, the incidence of autochthonous cases in Spain remained relatively stable between 2016 and 2019, with rates ranging from 0.85 to 0.90 cases per 100,000 inhabitants. In 2020, there was a decline, and in 2021 a slight increase [14]. The regions with the highest incidence during this period were the Community of Valencia (2.98), the Balearic Islands (2.97), and Murcia (1.08). In Catalonia, the incidence in 2019–2021 was 0.6 [14] and in Aragon 1.6 from 2000 to 2019 [15].

Several methods have been used to identify *Leishmania* parasites at different taxonomic levels, including for epidemiological studies [16,17]. Over the past 25 years, multilocus enzyme electrophoresis [18] has been the standard procedure for identifying *Leishmania* species and subspecies [19]. However, this analytical technique has some drawbacks, including insufficient discriminatory power to detect genetic diversity within *L. infantum* [19]. Currently, a wide range of DNA targets are used to study genetic diversity and phylogeny, such as the mini-exon [20,21], hsp70 gene [22], ITS-1 and ITS-2 regions [23,24,25,26], microsatellites [27,28], genes encoding antigens (cpb, gp63) [29,30], and kinetoplast DNA (kDNA) minicircles [26,31,32].

SNP and RFLP genotype analyses, the main techniques used to study intraspecific variation in kDNA, have revealed genetic variability in *L. infantum* from different endemic areas and reservoir hosts [2,19,26,33,34,35]. To date, 11 (G1–G11) and 20 (A–T) *L. infantum* genotypes have been identified using the SNP and RFLP methodologies, respectively. In the Mediterranean basin, these genotypes have been detected mainly in humans and dogs, but also in wildlife like foxes, rabbits, or rats, with some being shared across hosts (e.g., G1 and genotype B) and others found exclusively in humans (e.g., G9 and genotype T) or dogs (e.g., G4 and genotype S) [2,19,26].

The aim of the present study was to provide new insights into the intraspecific genetic variability of *L. infantum* from human and canine hosts in the Spanish Mediterranean region. Therefore, to further our understanding of the epidemiology of leishmaniasis, kDNA minicircles were analyzed as discriminative molecular markers.

## 2. Materials and Methods

### 2.1. Human and Dog Samples

A total of 26 samples were obtained from *Leishmania*-infected hosts in north-east Spain: ten humans (*n* = 21) from Catalonia and five dogs (*n* = 5) from Aragon. Of the human samples, collected in 2001–2002 [28], one was obtained from a cutaneous lesion and the rest (20) from bone marrow or buffy coats. Except for two individuals, all human patients were HIV-positive. Four of the 10 patients were followed up for periods ranging from six months to one year. After in vitro culture of the parasites present in the biological samples, DNA extraction was performed using the Chelex method [28]. The DNA was extracted with the informed consent of participants and approval from the ethics committees of the institutions. All dog samples were obtained from lymph node aspirates in 2020. DNA was extracted from the biological samples using the Roche High Pure PCR Template Preparation Kit following the manufacturer’s recommendations for each type of sample used.

### 2.2. PCR Amplification

PCR amplification of the kDNA from the mitochondrial minicircles was performed with primers MC1 (5′-GTTAGCCGATGGTGGTCTTG-3′) and MC2 (5′-CACCCATTTTTCCGATTTTG-3′) [36], amplifying a fragment of approximately 447 bp, including 405 bp of the variable region and 42 bp of the conserved region [2].

The PCR was carried out in volumes of 25 µL consisting of 2 µL of DNA and 23 µL of the reaction mixture containing 2.5 mM MgCl_2_, 0.2 mM dNTPs, 0.5 µM of each primer, 0.05 U/µL of RedTaq polymerase, and 1X buffer (Sigma-Aldrich, St. Louis, MO, USA). The thermocycling conditions to amplify the kDNA began with one cycle at 94 °C for 5 min, followed by 30 cycles at 94 °C for 30 s, 60 °C for 30 s, and 72 °C for 30 s. The final extension cycle was at 72 °C for 5 min.

To verify PCR amplification, the product was electrophoresed at 120 V for ~40 min on a standard 2% agarose gel. Finally, the product was visualized using a UV light transilluminator (Slite 140S).

### 2.3. Sequencing and Analysis of Single Nucleotide Polymorphisms

The amplified kDNA products were prepared for sequencing using the same primers as in the PCR amplification. Amplicons were previously purified using Wizard ^TM^ PCR prep (Promega, WI, USA). Sequencing was carried out by the Genomics Unit of the Scientific and Technological Centers of the University of Barcelona (CCiTUB). Sequences were edited using the BioEdit program and the consensus sequence was searched against the GenBank database using the nBLAST tool. The sequence KX098509 was chosen as a reference [26]. A multiple alignment was performed with the ClustalX2 program. Genetic distances (expressed in percentages) were calculated by the p-distance method with gamma distribution (G) [37] using MEGA11 software version 11.0.13.

### 2.4. Restriction Fragment Length Polymorphism Analysis

The variability of the kDNA sequences was analyzed by an in silico RFLP technique using the online Restriction Mapper program (www.restrictionmapper.org accessed on 30 April 2024). A panel of nine endonucleases (BglII, Bme1390I, DdeI, HpaII, RsaI, VspI, PstI, SfcI, and XapI) was used, and patterns based on fragment number and size allowed the sequences to be classified in RFLP genotypes [2,19,26].

### 2.5. Phylogenetic Analysis

To study the phylogenetic relationships between the SNP genotypes, a haplotype parsimony network was constructed using PopART [38], including all samples from the present study and those previously identified by Ortuño et al. [26]. The analysis was performed using the statistical parsimony procedure (95% parsimony connection limit) implemented in TCS [39]. Before constructing the network, SNP samples were processed using DnaSP V5.10.01 software [40].

To analyze the phylogenetic relationships between the RFLP genotypes, a binary matrix was generated from the presence (1) or absence (0) of each band in each genotype. All RFLP genotypes previously established by Cortes et al. [2], El Hamouchi et al. [19], and Ortuño et al. [26] were included, together with the new one described in the present study. Based on the resulting binary matrix, Neighbor-Net in SplitsTree version 5 was used to build a phylogenetic tree with the p-distance and equal angle methods [41]. The reliability of the tree was assessed by the bootstrap method with 1000 replications.

## 3. Results

### 3.1. Genotypes Obtained by SNP Analysis

The BLAST analysis of kDNA identified all sequences as *L. infantum*, with 97–100% identity with the reference sequence (KX098509). Insertions, deletions, and up to four SNPs were detected in 20 of the 26 kDNA sequences, with none in the remaining six. Sequences were grouped into seven SNP genotypes (G1, G2, G12–G15, and G17) (Table 1). DNA sequences of the minicircle kDNA corresponding to the seven identified genotypes were deposited in GenBank under the accession numbers PP848096-102. Ortuño et al. [26] previously established 11 SNP genotypes (G1–G11) in *L. infantum*, two of which were identified in the present study (G1 and G2). Additionally, we identified five new genotypes, which were named G12–G15 and G17, using the same nomenclature [26]. Considering isolates from relapses as different samples, the genotypes were found in the following frequencies: G13: 54%, G1: 23%, G2: 8%, and the rest, 4%.

The SNP genotypes identified in this study are listed in Table 2, along with the characteristics of the analyzed samples. Genotypes G1, G2, and G13 were observed in multiple individuals, while the others were only detected in single individuals. G13 stands out as the most common genotype, being found in five humans (*n* = 14). G1 was the only genotype found in both humans and dogs, whereas G12–G14 and G17 were exclusive to humans and G2 and G15 exclusive to dogs. Table S1 shows the genetic distances between each genotype.

### 3.2. Genotypes Obtained by RFLP Analysis

In silico RFLP analysis revealed five genotypes, four of which (B, F, N, and P) have been previously described by Cortes et al. [2] and Ortuño et al. [26]. The other genotype (W) is described here for the first time and is based on new restriction patterns obtained with the enzymes PstI (pattern II) and SfcI (pattern III) (Tables S2 and S3).

Considering isolates from relapses as different samples, genotypes were found at frequencies of 85% for B and 4% for the rest (Table 2). Apart from B, no genotype was detected in more than one sample. Genotype B was found in the two host types, whereas genotype N was found in a dog, and genotypes P, F, and W in humans.

### 3.3. Association between SNP and RFLP Genotypes

**Figure 1:**
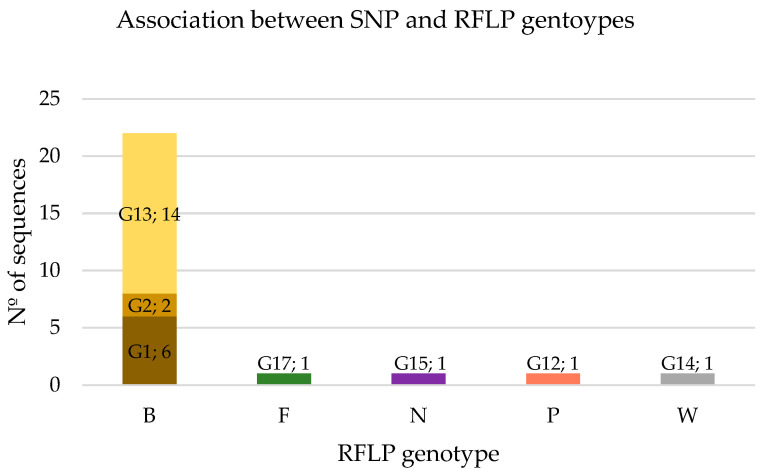
Number of SNP genotypes corresponding to each RFLP genotype. The SNP genotype and the number of corresponding sequences are indicated inside the bars of the graph. The total number of sequences corresponding to the RFLP genotype is shown in the vertical axis.

As shown in Figure 1, RFLP genotype B encompasses three SNP genotypes, G1 (27%), G2 (9%), and G13 (64%), which include two SNP variants (0 and 1) (Table 1). The other RFLP genotypes were associated with a single SNP genotype and therefore with different SNP variants (indicated the number of SNPs in brackets): F-G17 (4), N-G15 (1), P-G12 (1), and W-G14 (2).

For both methodologies (SNP and RFLP), no significant differences in genotypes were observed when comparing the samples collected from patients during follow-up (patients 1, 2, 3, and 6) (Table 2). In these patients, the detected genotypes corresponded to G13 and B, respectively. The only exception was the bone marrow sample of patient 2, where the identified genotypes were G14 and W, respectively, differing from the genotypes detected in the buffy coat sample of the same patient during the same period.

### 3.4. Phylogenetic Study

**Figure 2:**
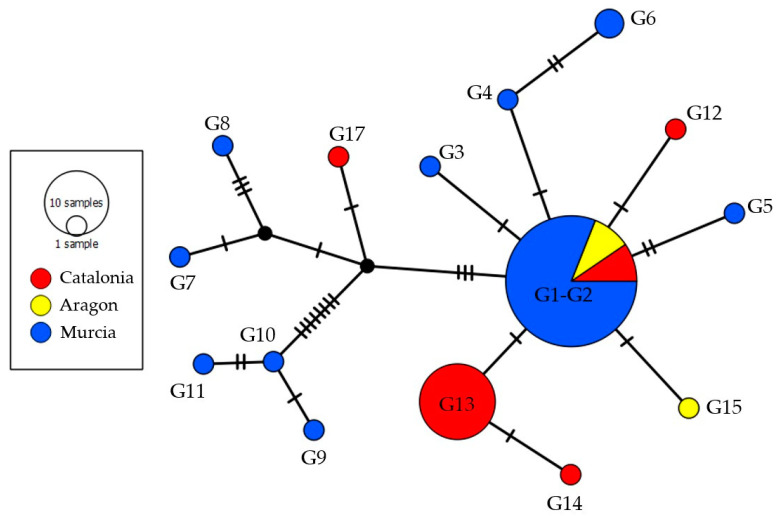
TCS network of *L. infantum* SNPs of kDNA minicircles from humans and dogs obtained in the present study and by Ortuño et al. [26] in humans, dogs, and wildlife. Hatch marks indicate mutations. Circle sizes are proportional to the number of shared individuals per haplotype.

The haplotype parsimony network (TCS) analysis, inferred from the hypervariable regions of kDNA minicircles of *L. infantum* and including the genotypes from Ortuño et al. [26], is shown in Figure 2. Except for G1 and G2, all genotypes were detected in a specific geographical area. Similarly, only G1, G2, and G6 shared a host type.

**Figure 3:**
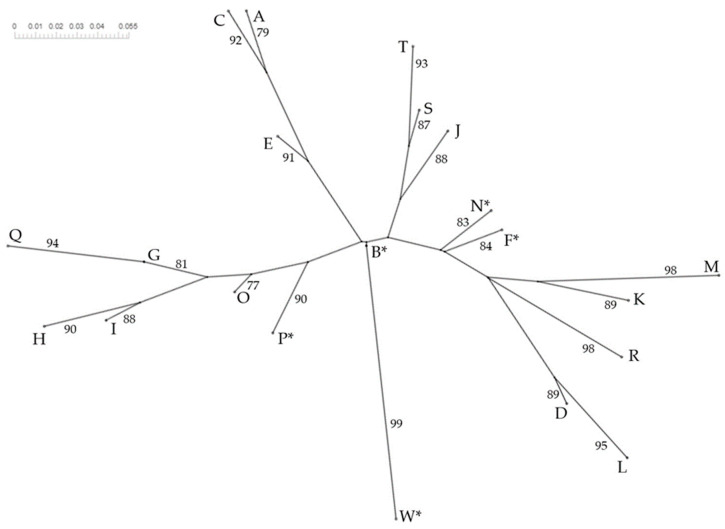
Neighbor-Net phylogenetic tree (neighbor-joining method) built up from kDNA-PCR-RFLP patterns of *Leishmania infantum* established in the present study and others [2,19,26]. * RFLP genotypes detected in the present study. Bootstrap values higher than 75 are given at the corresponding branches.

The Neighbor-Net phylogenetic tree constructed using a binary presence/absence matrix (Table S4) distinguishing several groups and showed the relationship between the RFLP genotypes described (A–W) by Cortes et al. [2], El Hamouchi et al. [19], Ortuño et al. [26], and in the present study (Figure 3).

## 4. Discussion

Advances in molecular biology have led to the development of various PCR-based methods for *Leishmania* typing [42]. In addition to being discriminative, they are simple to use and can even be directly applied to host biological tissue without the need for parasite isolation or culture [43,44,45,46]. In the present study, the hypervariable regions of kDNA minicircles of *L. infantum* were analyzed. Minicircle DNA is key for mitochondrial function in trypanosomatids, as it encodes guidance for RNA editing and plays a vital role in editing maxicircle mRNA transcripts encoding mitochondrial proteins [47]. As in previous studies [2,19,26], this genetic target proved highly discriminative and provided new insights into the genotypic distribution of *L. infantum* in a Mediterranean endemic area. The polymorphism observed in the kDNA analysis is due to the high sequence variability of minicircles, which have 10,000 copies/cell [48].

In this study, the SNP technique grouped 26 DNA sequences of *L. infantum* from two areas in the north-east of Spain into seven different genotypes (G1, G2, G12–G15, and G17), five of which are described here for the first time (G12–G15 and G17). It is worth highlighting the genetic proximity of most of the genotypes, which is apparent in the genetic distances between the SNP genotypes (Table S3). In a previous study by Ortuño et al. [26] in the south-east of Spain, 44 DNA sequences from *L. infantum* were grouped into 11 genotypes (G1–G11), only two of which (G1 and G2) were detected here (Figure 2). This difference in genotype profile could be related to the focal distribution of the disease, as hosts within a small geographical area infected with the same (or similar) SNP genotypes of *L. infantum* are likely to share a transmission cycle [26].

Four of the seven genotypes (57%) were identified in only one sample, a slightly lower percentage compared to Ortuño et al. (72%) [26]. Consistent with the findings of the latter study, G1 was the only genotype detected in both humans and dogs. Notably, in the south-east of Spain, although G2 was found in dogs and not in humans, as here, it was also present in all the wildlife species analyzed (brown bear, beech marten, rabbit, fox, genet, wolf, and rat) (Figure 2) [26]. Wildlife samples were not analyzed in the present study, but it would be of interest to include them in future research to shed more light on the role of wild reservoirs in the *Leishmania* life cycle.

Ortuño et al. [26] reported that although RFLP analysis was less discriminative than SNP genotyping, it was equally capable of grouping *L. infantum* variants specific to humans, dogs, and wildlife. In their study, RFLP analysis of kDNA revealed genotype B to be the most frequent (82%), being found in dogs, humans, and wildlife, whereas genotype F was detected exclusively in humans. In the present study, genotype B was also the most frequent, and likewise found in both dogs and humans. It was detected in 12 of the 15 studied hosts (80%) and in 85% of the samples (22/26). Furthermore, genotype B continued to be detected in samples collected from patients after clinical relapses. In other studies carried out in the Mediterranean basin, genotype B has been widely identified in both human and canine samples. The highest frequency of detection was in Morocco (69%) [19], followed by Portugal (30%), where it was the second-most common after genotype A [2]. As in the present study, genotype F has previously only been described in humans [2,19,26,49] and genotype N in dogs [2,19]. In contrast, genotype P is herein associated with the human host for the first time.

Also based on RFLP analysis, Risueño et al. [50] reported genotype B in seven *L. infantum*-infected foxes in the Spanish region of Murcia. Identification of the parasite in wildlife has stimulated debate about a sylvatic transmission cycle of *L. infantum*, which may occur independently of a domestic cycle maintained by dogs or interact with it [51]. As the main reservoir host in the domestic and peridomestic cycle of *L. infantum*, dogs are likely to be infected by other genotypes hitherto not associated with them. Among the dog samples from Aragon, four were associated with genotype B and one with genotype N.

The phylogenetic analysis of RFLP genotypes (Figure 3) supports the results of previous studies regarding *L. infantum* phylogenetic relationships [2,19,26,49], with additional knowledge provided by the identification of new RFLP genotypes. The range of *L. infantum* genetic variants observed in a geographical area associated with one or several hosts should be confirmed by further research with a higher number of samples from humans, dogs, and potential wildlife reservoirs, as well as from the parasite vectors.

Understanding the relationship between *L. infantum* genotypes and clinical manifestations or tissue tropism could be important in terms of disease epidemiology and the development of effective control strategies. In the present work, with one exception, genotype identification remained consistent in the samples taken from human patients following clinical relapses and analyzed after in vitro culture. However, it has been documented that long-term in vitro culture of kinetoplastid parasites may lead to the selection of a specific population differing from those of the sampled clinical tissue [2,52,53]. Recent research has found that *Leishmania* species acquire fitness advantages by adapting to culture conditions through diverse genetic mechanisms, suggesting that even minor genetic differences may have a significant impact on *Leishmania* adaptation to a given environment [54,55,56]. It is important to note that the human samples in the present study were analyzed after in vitro culture, whereas the genotypes in dogs were identified by DNA extraction directly from the tissue lesion. It would be of interest to compare the two approaches in both host types, also using other methodologies such as NGS-based techniques, as this would shed light on whether the dominant *L. infantum* genotype during the infection maintains its fitness during culture. This is particularly relevant, as infections might be caused by mixed populations or by parasites with different frequencies of minicircles [2].

## 5. Conclusions

This study of *L. infantum* genotypic distribution in endemic Mediterranean areas of north-east Spain confirmed the discriminative capacity of SNP and RFLP analysis of kDNA minicircles, indicating the suitability of these techniques for fingerprinting and strain monitoring. The most frequent SNP genotype was G13, detected herein for the first time and found exclusively in human samples. In the RFLP analysis, genotype B was predominant, identified in both humans and dogs. Additionally, the maintenance of genotypes over time was observed following clinical relapses. Overall, the results suggest a substantial intraspecific genetic diversity and a circulation of genetic variants between hosts, including humans and dogs. These findings offer valuable insights into the genetic diversity of *L. infantum* in Spain, with potential implications for disease epidemiology and the development of effective control measures.

## Figures and Tables

**Table 1:** Identified genotypes based on single-nucleotide polymorphisms (SNPs) of the kDNA, including the reference sequence KX098509. The variable positions of the nucleotides are shown with the corresponding substitutions, insertions, and deletions.

	Variable Nucleotide Positions												
SNP Genotypes	26	71	122	127	153	299	308	325	354	367	382	412	Ratio (%)
KX098509	A	C	C	—	A	A	A	G	T	A	—	C	
G1	.	.	.	.	.	.	.	.	.	.	.	.	23
G2	.	—	.	.	.	.	.	.	.	.	.	.	8
G12 *	.	.	.	.	G	.	.	.	.	.	.	.	4
G13 *	.	.	.	.	.	.	G	.	.	.	.	.	54
G14 *	.	.	.	.	.	G	G	.	.	.	.	.	4
G15 *	.	.	.	.	.	.	.	.	.	.	G	T	4
G17 *	G	.	A	T	.	.	.	—	C	G	.	.	4

SNP genotypes G1 and G2 were previously established by Ortuño et al. [26]. The new genotypes identified in the present study, G12–G15 and G17, are marked with an asterisk. Ratio: frequency of each genotype detected in the present work.

**Table 2:** Characteristics of the studied samples: host type, tissue type, and associated SNP and RFLP genotypes.

S.n.	I.n.	Host	Type of Tissue	SNP Genotype	RFLP Genotype
1	1 *	Human	Buffy coat	G13	B
2	1 *	Human	Buffy coat	G13	B
3	1 *	Human	Buffy coat	G13	B
4	1 *	Human	Buffy coat	G13	B
5	1 *	Human	Bone marrow	G13	B
6	2 *	Human	Buffy coat	G13	B
7	2 *	Human	Bone marrow	G14	W
8	2 *	Human	Buffy coat	G13	B
9	3 *	Human	Buffy coat	G13	B
10	3 *	Human	Skin	G13	B
11	4 *	Human	Bone marrow	G1	B
12	4 *	Human	Buffy coat	G1	B
13	5 *	Human	Bone marrow	G1	B
14	6 *	Human	Buffy coat	G13	B
15	6 *	Human	Buffy coat	G13	B
16	6 *	Human	Buffy coat	G13	B
17	6 *	Human	Buffy coat	G13	B
18	7 *	Human	Bone marrow	G13	B
19	8 *	Human	Bone marrow	G17	F
20	9	Dog	Lymph node	G2	B
21	10	Dog	Lymph node	G2	B
22	11	Dog	Lymph node	G15	N
23	12	Dog	Lymph node	G1	B
24	13	Dog	Lymph node	G1	B
25	14	Human	Bone marrow	G1	B
26	15	Human	Bone marrow	G12	P

S.n.: sample number; I.n.: individual number. * HIV-positive.

## Data Availability

The data presented in this study are available on request from the corresponding author.

## Supplementary Materials

The following supporting information can be downloaded at https://www.mdpi.com/article/10.3390/ani14121796/s1. Table S1: Estimates of genetic distance (%) between SNP-based genotypes using the p-distance model. Table S2: Patterns of enzymatic digestion of kDNA and corresponding fragment size (bp); Table S3: RFLP genotypes (A–W) and their corresponding restriction enzyme patterns; Table S4: Binary matrix of the presence (1) or absence (0) of bands in each RFLP genotype described by Cortes et al. [2], El Hamouchi et al. [19], Ortuño et al. [26], and in the present study.
