# Supplementary material for: Genetic Variability in Leishmaniasis-Causing Leishmania infantum in Humans and Dogs from North-East Spain
Source: Animals (Basel). 2024 Jun 15;14(12):1796. doi: 10.3390/ani14121796 (PMC11200389; doi:10.3390/ani14121796)
Supplement: Supplementary file 1 [file animals-14-01796-s001.zip › animals-3016155-supplementary.pdf]

Table S1: Estimates of genetic distance (%) between SNP-based genotypes, using the p-distance model.

	G1	G2	G12	G13	G14	G15	G17
**G1**	-						
**G2**	0.0	-					
**G12**	0.2	0.2	-				
**G13**	0.2	0.2	0.4	-			
**G14**	0.4	0.4	0.7	0.2	-		
**G15**	0.2	0.2	0.4	0.4	0.7	-	
**G17**	0.9	0.9	1.1	1.1	1.3	1.1	-

Table S2. Patterns of enzymatic digestion of kDNA and the corresponding fragment size (bp).

Restriction Enzymes	BglII		Bme1390I				DdeI			HpaII							RsaI				VspI			PstI		SfcI			XapI	
Patterns	**I**	II	**I**	II	III	IV	**I**	II	III	**I**	II	**III**	**IV**	V	VI	VII	**I**	II	III	IV	**I**	II	**III**	**I**	**II***	**I**	II	**III***	**I**	II
Fragments (bp)	447	258	411	288	447	324	319	419	240	410	287	447	350	324	229	352	253	210	447	253	161	170	310	298	447	224	294	224	347	347
		189	36	123		123	100	28	180	37	123		57	123	123	94	194	197		144	150	130	137	149		153	153	223	60	100
				36			28		28		37		37		58			40		48	136	90				70			40	
															37							50								

In bold, the enzymatic digestion patterns found in the present study; *New patterns of the enzymatic digestion described in this study for the first time.

Table S3: RFLP genotypes (A-W) and their corresponding restriction enzyme patterns.

Genotypes *RFLP*	BglII	Bme1390I	DdeI	HpaII	RsaI	VspI	PstI	SfcI	XapI
A	II	I	II	I	I	I	I	I	I
**B**	I	I	I	I	I	I	I	I	I
C	II	I	III	I	I	I	I	I	I
D	I	II	I	II	I	I	I	I	I
E	I	I	II	I	I	I	I	I	I
**F**	I	I	I	IV	I	I	I	I	I
G	I	I	I	I	II	II	I	I	I
H	II	I	I	I	II	II	I	I	I
I	II	I	I	I	I	II	I	I	I
J	I	I	I	I	IV	I	I	I	I
K	I	III	I	III	I	I	I	I	I
L	I	II	I	II	IV	I	I	I	I
M	I	III	I	IV	II	I	I	I	I
**N**	I	I	I	III	I	I	I	I	I
O	I	I	I	I	I	II	I	I	I
**P**	I	I	I	I	I	III	I	I	I
Q	I	I	I	I	II	II	I	I	II
R	I	IV	I	V	I	I	I	I	I
S	I	I	I	I	III	I	I	I	I
T	I	I	I	II	III	I	I	I	I
**W***	I	I	I	I	I	I	II**	III**	I

In bold, genotypes detected in the present study; *New RFLP genotype established in this study; **New restriction patterns.

Table S4. Binary matrix of the presence (1) or absence (0) of bands in each RFLP genotype described by Cortes et al. [2], El Hamouchi et al. [19], Ortuño et al. [26], and in the present study.

**Enzymes**	**BglII**		**Bme1390I**				**DdeI**			**HpaII**							**RsaI**				**VspI**			**PstI**		**SfcI**			**XapI**	
**Patterns**	**I**	**II**	**I**	**II**	**III**	**IV**	**I**	**II**	**III**	**I**	**II**	**III**	**IV**	**V**	**VI**	**VII**	**I**	**II**	**III**	**IV**	**I**	**II**	**III**	**I**	**II**	**I**	**II**	**III**	**I**	**II**
A	0	1	1	0	0	0	0	1	0	1	0	0	0	0	0	0	1	0	0	0	1	0	0	1	0	1	0	0	1	0
B	1	0	1	0	0	0	1	0	0	1	0	0	0	0	0	0	1	0	0	0	1	0	0	1	0	1	0	0	1	0
C	0	1	1	0	0	0	0	0	1	1	0	0	0	0	0	0	1	0	0	0	1	0	0	1	0	1	0	0	1	0
D	1	0	0	1	0	0	1	0	0	0	1	0	0	0	0	0	1	0	0	0	1	0	0	1	0	1	0	0	1	0
E	1	0	1	0	0	0	0	1	0	1	0	0	0	0	0	0	1	0	0	0	1	0	0	1	0	1	0	0	1	0
F	1	0	1	0	0	0	1	0	0	0	0	0	1	0	0	0	1	0	0	0	1	0	0	1	0	1	0	0	1	0
G	1	0	1	0	0	0	1	0	0	1	0	0	0	0	0	0	0	1	0	0	0	1	0	1	0	1	0	0	1	0
H	0	1	1	0	0	0	1	0	0	1	0	0	0	0	0	0	0	1	0	0	0	1	0	1	0	1	0	0	1	0
I	0	1	1	0	0	0	1	0	0	1	0	0	0	0	0	0	1	0	0	0	0	1	0	1	0	1	0	0	1	0
J	1	0	1	0	0	0	1	0	0	1	0	0	0	0	0	0	0	0	0	1	1	0	0	1	0	1	0	0	1	0
K	1	0	0	0	1	0	1	0	0	0	0	1	0	0	0	0	1	0	0	0	1	0	0	1	0	1	0	0	1	0
L	1	0	0	1	0	0	1	0	0	0	1	0	0	0	0	0	0	0	0	1	1	0	0	1	0	1	0	0	1	0
M	1	0	0	0	1	0	1	0	0	0	0	0	1	0	0	0	0	1	0	0	1	0	0	1	0	1	0	0	1	0
N	1	0	1	0	0	0	1	0	0	0	0	1	0	0	0	0	1	0	0	0	1	0	0	1	0	1	0	0	1	0
O	1	0	1	0	0	0	1	0	0	1	0	0	0	0	0	0	1	0	0	0	0	1	0	1	0	1	0	0	1	0
P	1	0	1	0	0	0	1	0	0	1	0	0	0	0	0	0	1	0	0	0	0	0	1	1	0	1	0	0	1	0
Q	1	0	1	0	0	0	1	0	0	1	0	0	0	0	0	0	0	1	0	0	0	1	0	1	0	1	0	0	0	1
R	1	0	0	0	0	1	1	0	0	0	0	0	0	1	0	0	1	0	0	0	1	0	0	1	0	1	0	0	1	0
S	1	0	1	0	0	0	1	0	0	1	0	0	0	0	0	0	0	0	1	0	1	0	0	1	0	1	0	0	1	0
T	1	0	1	0	0	0	1	0	0	0	1	0	0	0	0	0	0	0	1	0	1	0	0	1	0	1	0	0	1	0
W*	1	0	1	0	0	0	1	0	0	1	0	0	0	0	0	0	1	0	0	0	1	0	0	0	1	0	0	1	1	0

*New RFLP genotype established in this study.
